# Supplementary material for: The IRE1/XBP1 signaling axis promotes skeletal muscle regeneration through a cell non-autonomous mechanism
Source: eLife. 2021 Nov 23;10:e73215. doi: 10.7554/eLife.73215 (PMC8635982; doi:10.7554/eLife.73215)
Supplement: Source data 1. [file elife-73215-supp1.zip › Source data-1 eLife.pptx]

## Slide 1
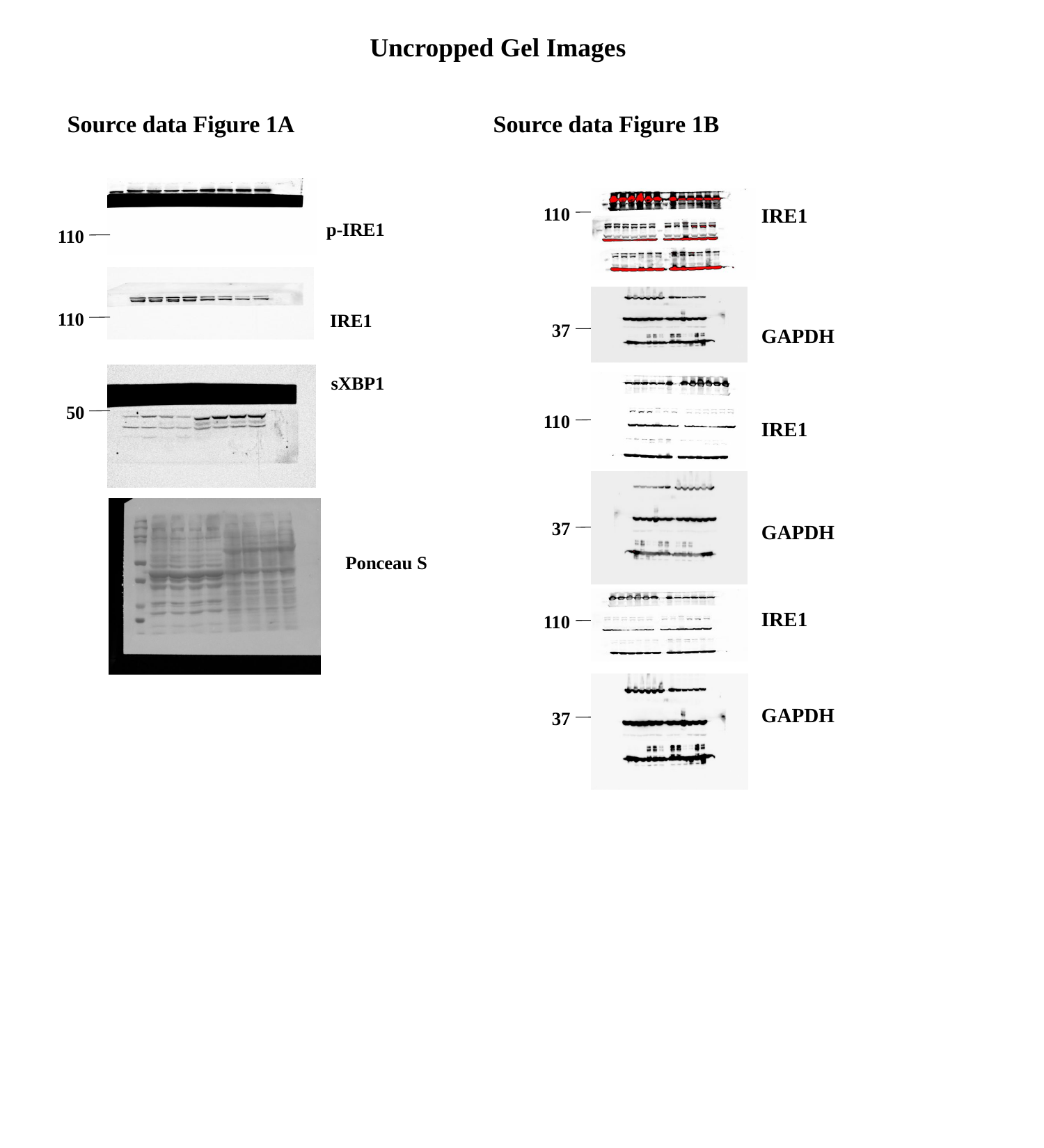

Uncropped Gel Images
Source data Figure 1A
Source data Figure 1B
110
IRE1
37
GAPDH
110
IRE1
37
GAPDH
IRE1
110
GAPDH
37
p-IRE1
110
110
IRE1
sXBP1
50
Ponceau S

## Slide 2
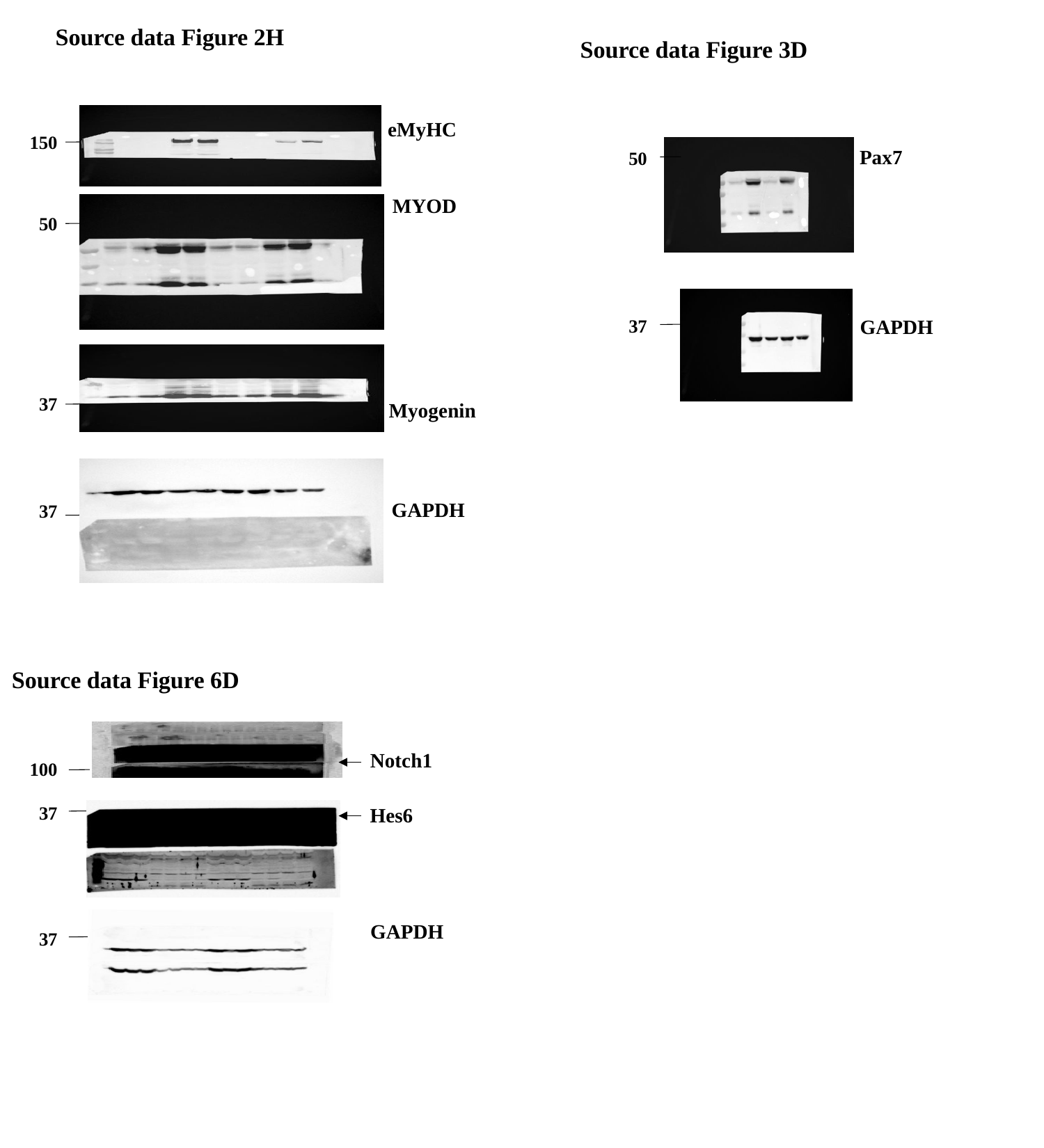

Source data Figure 2H
Source data Figure 3D
eMyHC
150
Pax7
50
MYOD
50
GAPDH
37
37
Myogenin
GAPDH
37
Source data Figure 6D
Notch1
100
37
Hes6
GAPDH
37

## Slide 3
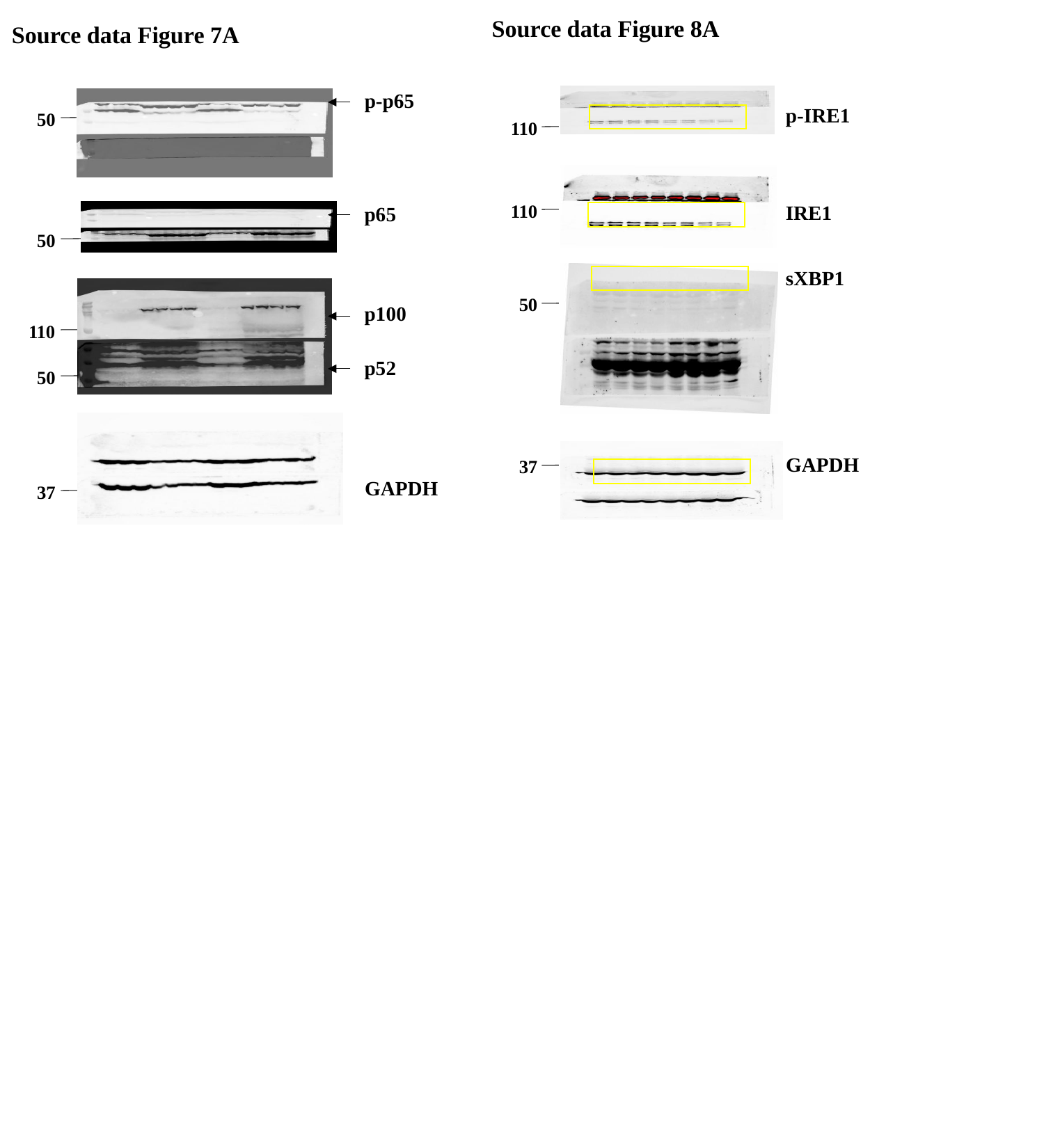

Source data Figure 8A
Source data Figure 7A
p-p65
p-IRE1
110
110
IRE1
sXBP1
50
GAPDH
37
50
p65
50
p100
110
p52
50
GAPDH
37
