# Supplementary material for: The IRE1/XBP1 signaling axis promotes skeletal muscle regeneration through a cell non-autonomous mechanism
Source: eLife. 2021 Nov 23;10:e73215. doi: 10.7554/eLife.73215 (PMC8635982; doi:10.7554/eLife.73215)
Supplement: Peer review history

# Editor report: Editor's evaluation

**DOI:** 10.7554/eLife.73215.sa0

**Authors:** Christopher Cardozo

**Affiliations:** United States

The manuscript evaluated an under appreciated aspect of tissue regeneration which is the need to synthesize and assemble huge amounts of contractile machinery and supporting structures and metabolic enzymes. The use of a muscle creatine kinase germline knockout of the gene encoding Ire1/Ern1 shifts the focus from traditional studies of tissue repair toward the regenerating myofibers. The findings shed new light on mechanisms of regeneration of healthy myofibers after severe tissue injury and suggest interplay between these fibers and muscle satellite cells though mechanisms remain to be elucidated.

# Decision letter

**DOI:** 10.7554/eLife.73215.sa1

**Affiliations:** United States; United States

In the interests of transparency, eLife publishes the most substantive revision requests and the accompanying author responses.

**Decision letter after peer review:**

Thank you for submitting your article "IRE1/XBP1 signaling promotes skeletal muscle regeneration through a cell non-autonomous mechanism" for consideration by *eLife*. Your article has been reviewed by 3 peer reviewers, including Christopher Cardozo as the Reviewing Editor and Reviewer #3, and the evaluation has been overseen by Mone Zaidi as the Senior Editor.

The reviewers have discussed their reviews with one another, and the Reviewing Editor has drafted this to help you prepare a revised submission.

Essential revisions:

1. The results of these genetic experiments need to be reconciled with those of a prior report showing that inhibition of the PERK arm of the unfolded protein response or genetic ablation of Perk1 inhibits muscle regeneration while inhibition of Irk1 does not. Differences in conclusions regarding requirements for Xbp1 between the current manuscript and the same prior report from this group should also be discussed.

2. Please clarify the rationale for the studies presented in Figure 8 in which mdx mice are crossed with IREmKO mice.

3. Please acknowledge and discuss limitations of knockout approaches and comment on the need for gain of function studies in the future.

4. Please discuss your findings in relationship to those recently reported by He et al. (PMC8409588) using a Pax7 promotor to ablate IRE1.

5. Please discuss purity of satellite cells (Figure 3E) and caveats for interpreting this experiment.

6. Please expand on the rational for performing the double injury experiment in Results. Please quantify data from Figure 1H.

7. Please expand on the discussion of the logic for concluding that gene expression for altered Notch signaling indicates non-cell autonomous mechanisms; please discuss alternative interpretations.

8. One assumes that unfolded protein responses are increased during tissue regeneration due to the huge increase in demand for synthesis, processing, secretion and signaling via the diverse array of proteins deposited in extracellular matrix during tissue repair, and synthesis of an entirely new set of contractile proteins, cytoskeletal proteins, membrane proteins and machinery for homeostasis (ion channels, adhesion molecules, dystrophin, and others) and that activation of UPR is a physiological response that assures that cells participating in regeneration continue to function efficiently in the face of these many demands. A brief paragraph on this biology from the teleological perspective would strengthen the manuscript.

9. Discuss caveats in interpreting the data on XBP1 with regard to effects of the knockout on satellite cell proliferation; add data on this outcome if feasible (Ki67 immunostaining).

10. Please discuss relationships between the Notch and NF-κB pathway studies. Expand on the rational for adding analysis of NF-κB to the outcomes evaluated.

11. Please respond to the following comments and revise the manuscript as appropriate:

– Authors did look at different time points (day 5 and day 14) in figure 1, the rest of the data were collected from the one-time point. This is not enough to capture and dynamic changes of muscle stem cell myogenic response.

– Skeletal muscles are multinucleated, cross-sectional analyses of 10-15 micron muscle sections will not provide a full understanding of changes in myogenesis. In addition, these are myofiber-specific knockouts of IRE1/XBP1 using MCK Cre. Each nucleus in muscle could have a different unfolded protein response and transcriptional regulation during muscle regeneration. Especially, muscle stem cell-derived myonuclei vs. other uninjured myonuclei.

– Edu+ nuclei should be normalized to total nuclei. More importantly, in Figure 4A, I am not sure why fused myonuclei (central nuclei from muscle stem cells in myofiber) are Edu+. These are post-mitotic nuclei. Authors should test whether these are non-specific binding. Muscle fibers have autofluorescence in the green fluorescence (488nm) emission wavelength.

*Reviewer #1:*

In this manuscript, authors present a thorough description of downstream effects on muscle regeneration following disruption of IRE1/XBP1 activity. However, in its current form, the manuscript falls short of presenting a compelling and cohesive mechanism by which these effects occur.

Strengths of this manuscript include comprehensive histological analyses of skeletal muscle regeneration together with assessment of satellite cell fate. The use of muscle-specific KO mice is also a strength as is the a priori power analysis, which enhances the rigor of the work. The statistical analyses used are appropriate. The writing is clear and easy to follow.

One of the major weaknesses of this work is the use exclusively of loss-of-function paradigms. Given the critical role of the ER in protein synthesis and folding, it is not surprising that disruption of this system would drastically affect regeneration after an acute injury. The manuscript would be considerably strengthened by the addition of gain-of-function or rescue studies, and specifically as they pertain to the studies implicating Notch and/or NF-κB as downstream mediators of the effect of IRE1 on satellite cell proliferation.

It is interesting that the investigators identified activation of XBP1, but not the RIDD mechanism, as downstream of the effect of IRE1 on skeletal muscle regeneration. However, the data provided to rule out the RIDD mechanism are limited to gene expression analyses. Given that previous work has implicated RIDD in the regulation of muscle regeneration by IRE1, additional studies are needed to support the current authors' conclusions.

It is not clear why the investigators chose to perform a double injury experiment in Figure 1. The double injury data in Figure 1H, I are not quantified, so these images are not of high significance. Also, grip strength testing is performed in the mdx mice studies of Figure 8, but not in Figure 1. The addition of functional measures to Figure 1 would be valuable.

It is not clear to me how the findings of Notch signaling fit relate to the NFk-B findings and, as currently presented, the two findings seem disconnected. Similarly, satellite cell proliferation in the XBP1 muscle KO mice is not quantified, which would be helpful for supporting the investigators' overall proposed mechanism.

The rationale for the studies presented in Figure 8 in which mdx mice are crossed with IREmKO mice is not clear, nor is the interpretation of findings that the increased IRE1 in mdx mice, when reversed, aggravates the pathogenic phenotype.

It would be helpful if the investigators would provide a more thorough discussion of the physiological relevance of the current findings, which utilize a myofiber-specific KO of IRE, versus the previous work by He et al. in which IRE is knocked out specifically in myoblasts.

Comments for the authors:

This reviewer appreciates the extensive analyses that have been performed and that are presented in this manuscript. The authors are also to be commended for inclusion of the negative findings (e.g., the lack of effect on Wnt signaling). However, in its current form, many of the data presented describe the phenotype of the KO mice. The science would be strengthened by mechanistic interrogation of the hypothesis proposed.

I also recommend submission of full western blots as Supplementary files. The western blots shown in Figure 6 are of poor quality. Along these lines, for greater transparency, the graphs would be strengthened if changed to include individual data points.

Authors have not included a statement about whether investigators performing analyses were blinded to the treatment group, which is important as many of the analyses performed may be subject to bias.

Please provide information regarding age and sex of the animals used.

*Reviewer #2:*

The manuscript by Roy et al. demonstrates that IRE1/XBP1 signaling plays a significant role in muscle regeneration. Given that mature skeletal muscle fibers contain high concentrations of contractile and structural proteins, quality control mechanisms to maintain protein homeostasis are crucial. In this study, the authors used a loss of function approaches to test the role of IRE1 and its downstream transcriptional factor, XBP-1 following acute muscle injury and chronic muscular dystrophy model in mdx mice. However, due to the limitations in the experimental approach and study design, reported data do not support the authors' conclusions and alternative explanations can be identified. Overall, the authors do not provide mechanistic insights on how IRE-1 affecting muscle stem cell dynamics.

Comments for the authors:

The manuscript by Roy et al. reports interesting observations on how IRE1/XBP1 signaling affects acute and chronic myogenic response by satellite cells. Overall, given the importance of proteostasis in muscle, this is an important area of research in muscle biology. However, there several shortcomings that reduced enthusiasm for this study.

1. Experimental design – Authors did look at different time points (day 5 and day 14) in figure 1, the rest of the data were collected from the one-time point. This is not enough to capture and dynamic changes of muscle stem cell myogenic response.

2. Skeletal muscles are multinucleated, cross-sectional analyses of 10-15 micron muscle sections will not provide a full understanding of changes in myogenesis. In addition, these are myofiber-specific knockouts of IRE1/XBP1 using MCK Cre. Each nucleus in muscle could have a different unfolded protein response and transcriptional regulation during muscle regeneration. Especially, muscle stem cell-derived myonuclei vs. other uninjured myonuclei.

3. Edu+ nuclei should be normalized to total nuclei. More importantly, in Figure 4A, I am not sure why fused myonuclei (central nuclei from muscle stem cells in myofiber) are Edu+. These are post-mitotic nuclei. Authors should test whether these are non-specific binding. Muscle fibers have autofluorescence in the green fluorescence (488nm) emission wavelength.

4. Overall, the link between how muscle-specific knockout of IRE1 influences muscle stem cell myogenesis is weak. A snapshot of gene expression changes does not provide convincing evidence of non-cell-autonomous interactions.

5. Figure 3E, these are not a pure population of satellite cells that are >95% Pax7+. An additional positive satellite cell surface marker or different gating strategy should be used.

*Reviewer #3:*

This manuscript examines the role of Ire1, a protein involved in the unfolded protein response, in the proliferation and regenerative abilities of skeletal muscle satellite cells. A model of skeletal muscle regeneration following injection of barium chloride into the left tibialis anterior muscle (TA) with injection of normal saline into the right TA serving as the control. Phospho-Ire1 was increased by muscle injury. The role of Ire1 in muscle regeneration was tested in mice with a germline conditional deletion of Ire1 under a muscle creatine kinase promotor. The conditional Ire1 knockout diminished myofiber size out to at least day 14 after injury, reduced numbers of Pax7(+) nuclei in regenerating muscle, and reduced numbers of EdU(+) nuclei. Biochemical evidence of lower expression of Notch 1 and a downstream target, Hes6, in regenerating tissues is presented. The conditional knockout of Ire1 worsened grip strength in mdx mice. Taken together, the data support a role for unfolded protein response signaling through Ire1 in the ability of skeletal muscle satellite cells to proliferate and fuse during tissue repair.

Caveats to interpreting the data are universal to the field and include unanticipated effects of the germline deletion of Ire1 on muscle biology and the small N of some of the experiments.

The results of these genetic experiments need to be reconciled with those of a prior report showing that inhibition of the PERK arm of the unfolded protein response or genetic ablation of Perk1 inhibits muscle regeneration while inhibition of Irk1 does not. Differences in conclusions regarding requirements for Xbp1 between the current manuscript and a prior report from this group should also be discussed.

Additional details about some of the methods for morphometry should be added.

Comments for the authors:

The data on body and muscle weights showing no difference between IRE1(fl/fl) and Ire1 mKO mice should be shown either in supplemental materials or though a link to a resource on figshare or other online data repositories.

In the sentence on page 8 that states "with injured myofibers during muscle regeneration". My understanding is that barium chloride causes osmotic rupture of many myofibers. I thought it would be more accurate to simply say "fusion of satellite cells with regenerating myofibers".

Please define DSHB (Methods, Immunofluorescence).

[Editors' note: further revisions were suggested prior to acceptance, as described below.]

Thank you for resubmitting your work entitled "IRE1/XBP1 signaling promotes skeletal muscle regeneration through a cell non-autonomous mechanism" for further consideration by *eLife*. Your revised article has been evaluated by Mone Zaidi as the Senior Editor, and a Reviewing Editor.

The manuscript has been improved but there is one remaining issue that needs to be addressed, as outlined below:

I noticed that during revisions, Ire1 was replaced with Ern1. My understanding is that these are two designations for the same gene. Given that Ire1 is used in relevant published literature it makes sense to include this designation in the title and introduction. To aid the reader, please clarify in the text that IRE1 and Ern1 refer to the same gene..

# Reply: Author response

**DOI:** 10.7554/eLife.73215.sa2

Essential revisions:

1. The results of these genetic experiments need to be reconciled with those of a prior report showing that inhibition of the PERK arm of the unfolded protein response or genetic ablation of Perk1 inhibits muscle regeneration while inhibition of Irk1 does not. Differences in conclusions regarding requirements for Xbp1 between the current manuscript and the same prior report from this group should also be discussed.

This is an important suggestion. In our previously published report about the role of PERK in skeletal muscle regeneration, we had deleted Perk or Xbp1 in the satellite cells and the effects were cell-autonomous. In the present manuscript, we studied the role of IRE1 and XBP1 in differentiated myofibers where we deleted Ire1 and Xbp1 gene using muscle creatine kinase (MCK)-Cre line. MCK promoter is expressed in differentiated muscle cells only, not in muscle progenitor/satellite cells. We have now discussed this issue in the “Discussion” section of our manuscript (Page # 15, in first paragraph).

2. Please clarify the rationale for the studies presented in Figure 8 in which mdx mice are crossed with IREmKO mice.

All the muscle regeneration studies (in Figures 1-7) in our manuscript were performed using a model of acute skeletal muscle injury. The mdx mice is a model of chronic muscle degeneration and regeneration. Therefore, to study the role of IRE1 in myofibers during chronic muscle injury and regeneration, we crossed IRE1^mKO^ mice with mdx mice. We have now clearly explained this point in the Result section of the manuscript (Page # 12, last paragraph).

3. Please acknowledge and discuss limitations of knockout approaches and comment on the need for gain of function studies in the future.

We have now discussed this point in the “Discussion” section of the manuscript (Page # 18 and 19).

4. Please discuss your findings in relationship to those recently reported by He et al. (PMC8409588) using a Pax7 promotor to ablate IRE1.

He et al. used MyoD1-Cre line (active in myoblasts) not the Pax7 promoter line. We acknowledge that it is important to dissect the role of IRE1 in muscle progenitor cells. This has been further discussed in the “Discussion” section of the manuscript (Page # 16, first paragraph).

5. Please discuss purity of satellite cells (Figure 3E) and caveats for interpreting this experiment.

The FACS analysis in Figure 3E is done as another approach to confirm that myofiber-specific ablation of IRE1/ERN1 inhibits satellite cell abundance in injured myofibers. There is always a possibility that the satellite cell population identified by FACS may contain some other cell types as well. However, our results with FACS approach support the immunohistochemical (Figure 3A and 3B) and biochemical studies (Figure 3C and 3D) about the effect of myofiber-specific deletion of IRE1α on satellite cell number in regenerating muscle.

6. Please expand on the rational for performing the double injury experiment in Results. Please quantify data from Figure 1H.

We have now provided additional justification for double injury experiment. We have provided quantification of the data of Figure 1H in the new Figures 1I and 1J (Page # 6, second paragraph).

7. Please expand on the discussion of the logic for concluding that gene expression for altered Notch signaling indicates non-cell autonomous mechanisms; please discuss alternative interpretations.

Since we do not have concrete data about this aspect, we have removed the presumption that alteration in Notch signaling is indicative of non-cell autonomous mechanisms. Indeed, it is possible that Notch ligands and receptors are expressed in two different cells of the same cell type within an organ. In addition, factors produced by injured myofibers may also influence Notch signaling in a paracrine fashion. We have now specifically mentioned this aspect in the Discussion section of the manuscript (Page # 17, first paragraph).

8. One assumes that unfolded protein responses are increased during tissue regeneration due to the huge increase in demand for synthesis, processing, secretion and signaling via the diverse array of proteins deposited in extracellular matrix during tissue repair, and synthesis of an entirely new set of contractile proteins, cytoskeletal proteins, membrane proteins and machinery for homeostasis (ion channels, adhesion molecules, dystrophin, and others) and that activation of UPR is a physiological response that assures that cells participating in regeneration continue to function efficiently in the face of these many demands. A brief paragraph on this biology from the teleological perspective would strengthen the manuscript.

This is a very important point and has been now added to the “Introduction” section of the manuscript (Page # 4).

9. Discuss caveats in interpreting the data on XBP1 with regard to effects of the knockout on satellite cell proliferation; add data on this outcome if feasible (Ki67 immunostaining).

We do not have Ki67 immunostaining results for this figure. However, we have now specifically mentioned in the Results section that further experimentation is needed to determine whether XBP1 signaling in myofibers affect proliferation or survival of satellite cells in injured muscle microenvironment (Page # 10, second paragraph).

10. Please discuss relationships between the Notch and NF-κB pathway studies. Expand on the rational for adding analysis of NF-κB to the outcomes evaluated.

We thank the reviewer for this important point about Notch and NF-κB signaling. We have now added a few sentences and references about signaling cross-talk between Notch and NF-κB pathway in the Results (Page # 11, 12) and the Discussion (Page # 17) sections of the manuscript.

11. Please respond to the following comments and revise the manuscript as appropriate:

– Authors did look at different time points (day 5 and day 14) in figure 1, the rest of the data were collected from the one-time point. This is not enough to capture and dynamic changes of muscle stem cell myogenic response.

We agree that we have performed histological analysis at day 5 and day 14. Moreover, we have also analyzed muscle regeneration after double injury (second injury after 21 days of first injury). We have chosen day 5 for satellite cell analysis because at this time the number of satellite cell peaks and muscle is undergoing active regeneration. Therefore, it is easy to capture the changes in satellite cell response at day 5. Please note that in vivo EdU incorporation in regenerating myofiber was also performed at day 14 after injury.

– Skeletal muscles are multinucleated, cross-sectional analyses of 10-15 micron muscle sections will not provide a full understanding of changes in myogenesis. In addition, these are myofiber-specific knockouts of IRE1/XBP1 using MCK Cre. Each nucleus in muscle could have a different unfolded protein response and transcriptional regulation during muscle regeneration. Especially, muscle stem cell-derived myonuclei vs. other uninjured myonuclei.

We agree with the reviewer that cross-sectional analyses may not provide full understanding of the myogenesis. However, we have performed many other experiments including Western blot, QRT-PCR, and FACS analysis where whole muscle tissue was used. Results of our histological and biochemical analysis are highly consistent. In addition, we would like to clarify that for our experiments we ensure that > 90% of TA muscle is injured after injection of BaCl_2_ and therefore our analysis included mostly regenerating tissue.

– Edu+ nuclei should be normalized to total nuclei. More importantly, in Figure 4A, I am not sure why fused myonuclei (central nuclei from muscle stem cells in myofiber) are Edu+. These are post-mitotic nuclei. Authors should test whether these are non-specific binding. Muscle fibers have autofluorescence in the green fluorescence (488nm) emission wavelength.

We have also now provided percentage of EdU^+^ nuclei in total nuclei in the TA muscle sections (Figure 4D). These is no non-specific binding because not all the nuclei were EdU^+^ in the sections. We had given intraperitoneal injection of EdU at day 3 post-injury. EdU is a thymidine analogue which is incorporated into the DNA of dividing cells. Satellite cells that became activated and entered the cell cycle incorporate EdU. Because myonuclei are postmitotic, EdU^+^ nuclei represent satellite cells that became “activated” and have entered the cell cycle. Since only muscle progenitor cells fuse with injured myofibers, the EdU+ nuclei in myofibers is a representation of satellite cell activation/proliferation and fusion. This is a standard approach to measure the proliferation and fusion of satellite cells with regenerating skeletal muscle of mice.

[Editors' note: further revisions were suggested prior to acceptance, as described below.]

The manuscript has been improved but there is one remaining issue that needs to be addressed, as outlined below:

I noticed that during revisions, Ire1 was replaced with Ern1. My understanding is that these are two designations for the same gene. Given that Ire1 is used in relevant published literature it makes sense to include this designation in the title and introduction. To aid the reader, please clarify in the text that IRE1 and Ern1 refer to the same gene..

We used Ern1 in place of IRE1 as suggested by an editorial support staff of *eLife* journal. Ern1 is the gene name of IRE1α and therefore we used Ern1 to refer floxed mice (i.e. Ern1fl/fl) and conditional knockout (Ern1cKO). As you also noted that IRE1 is the most commonly used name of this gene in ER stress field. However, we agree that it is important to specifically mention that IRE1 and Ern1 refer to the same gene. We have now specially mentioned this in the Abstract (page # 2), Introduction (page # 3), and Results (Page # 5) sections of our manuscript.
